# Effects of L-Carnitine on the sperm parameters disorders, apoptosis of spermatogenic cells and testis histopathology in diabetic Rats

**DOI:** 10.18502/ijrm.v17i5.4600

**Published:** 2019-06-26

**Authors:** Tahereh Mardanshahi, Nourollah Rezaei, Zohreh Zare, Majid Malekzadeh Shafaroudi, Hamidreza Mohammadi

**Affiliations:** ^1^ Department of Anatomy and Cell Biology, Faculty of Medicine, Mazandaran University of Medical Sciences, Sari, Iran.; ^2^ Immunogenetics Research Center (IRC), Faculty of Medicine, Mazandaran University of Medical Sciences, Sari, Iran.; ^3^ Department of Toxicology and Pharmacology, Faculty of Pharmacy, Mazandaran University of Medical Sciences, Sari, Iran.

**Keywords:** Diabetes, L-carnitine, Sperm, Apoptosis, Testis.

## Abstract

**Background:**

Diabetes mellitus affects male reproductive system that is known to cause male infertility.

**Objective:**

The aim of the present study was to assess the effects of L-carnitine (LC) on sperm parameters, apoptosis of spermatogenic cells and testis histopathology in Streptozotocin-induced diabetic Rats.

**Materials and Methods:**

The study was carried out on 36 male Wistar adult rats (220 ± 30 gr) randomly divided into six groups (*n* = 6/each). 1 (Control); 2 (LC 100 mg/kg); 3 (Diabetic); 4, 5, and 6 (Diabetic + LC 50 or 100 or 200 mg/kg, respectively). Daily injections were administered intraperitoneally for 48 days. Then, rats were sacrificed, left testis and epididymis were harvested for sperm analysis and histopathology, morphometric and spermatogenesis assessments, and Tunnel assay.

**Results:**

L-carnitine in group 6 significantly decreased blood glucose level (p < 0.01) in comparison with group 3. L-carnitine in groups 5 and 6 significantly (p < 0.001) and dose-dependently increased the count, motility, viability, maturity, and chromatin quality of sperm and decreased the abnormal morphology of sperm in comparison with group 3. In groups 4, 5, and particularly 6, in comparison with group 3, there has been a significant difference in the increase of seminiferous tubule diameter, germinal epithelium height (p < 0.001), maturity quality of the seminiferous tubules (p < 0.001), decrease apoptosis of spermatogenic cells (p < 0.001), and testis tissue histopathological complications.

**Conclusion:**

The data obtained from the present study suggest that in the diabetic rats, LC decreases serum glucose level, improves the diameter and thickness of the epithelium of spermatogenic cells, reduces germ cells' apoptosis, and improves epididymal sperm parameters. Therefore, it seems that LC plays an effective role in diabetes-induced infertility.

## 1. Introduction

Diabetes mellitus (DM) is one of the most common endocrine disorders resulting from a diminished insulin secretion or insulin action or both. It is the fastest growing worldwide and one of the leading causes of deaths in the world. The epidemiological studies indicate that 10% of the world's population is affected by this disease (1). Diabetes can lead to complications in different body organs including the blood vessels, eyes, kidneys and nerves. One of the organs affected by diabetes is a male reproductive system, to such an extent that 90% of diabetic patients suffer from various genital functions disorders (2). The most important ones are spermatogenesis disorder, an increase of apoptosis in spermatogonium and spermatocyte cells, and reduction of sex hormones level. Diabetes also affects spermatogenesis including a decrease in sperm count, motility, and increase of abnormal sperms; also diabetes significantly decreases seminiferous tubules diameter and the weight of testis and body (3, 4). Gunely and co-worker studying the testicular damage in diabetic rats noticed the reduction of seminiferous tubules diameter and increased apoptosis (5). Rashidi and co-workers found that diabetes increases blood glucose levels, reduce spermatogonia and Sertoli cells in spermatogenesis process, even of sperm count and motility, and increases abnormal morphology of sperm (6).

In recent years, many studies have tried to find chemical and herbal drugs to resolve infertility in diabetic patients. One of the chemical compounds focused by the researchers is carnitine antioxidant (3-hydroxy-4-N-trimethylamino butyric acid) obtained from the meat and dairy products. L-carnitine (LC) was extracted from the bovine muscle for the first time in 1905. It is a water-soluble, quaternary amine synthesized from lysine and methionine, stored in skeletal muscles, heart, brain, and testis. L-carnitine are found in epididymis and sperm, its concentration is about 2000 times higher than in plasma. The sperm LC is important in lipid metabolism that facilitates long-chain fatty acids' β-oxidation in mitochondria and is necessary for energy production. It acts as a substantial non-enzymatic antioxidant, that protects the cell, mitochondrial membrane, and DNA integrity against free oxygen radicals (7). Several studies have been performed to evaluate the effect of carnitine on infertile men, indicating the improvement of sperm fertility, count, and motility. L-carnitine is effective in improving fertility (8, 9). The study of Lenzi and co-worker on 100 idiopathic infertile men indicates the effectiveness of LC in increasing semen quality, particularly sperm motility (10). However, LC's effect on diabetes-induced damage on sperm parameters and testicular structure has not been studied in diabetic patients.

Therefore, the aim of the present study was to investigate LC's effects on sperm parameters' disorders, spermatogenic cells apoptosis, and testis histopathologic in Streptozotocin (STZ)-induced diabetic adult Wistar Rats. It is hoped that the data obtained from this study could be a guideline in improving diabetes-induced complications on sperm parameters and testicular structure and be effective in infertility treatment.

## 2. Materials and Methods

### Animals

In this experimental study, 36 adult male Wistar rats (8–10 wk, 220 ± 30 gr) were taken from the animal center of Mazandaran University of Medical Sciences and kept under the standard conditions.

### Diabetes induction

A single intraperitoneal (IP) of freshly prepared STZ was injected (60 mg/kg in 0.1 M sodium citrate buffer, PH = 4.6), (STZ, S0130-500 MG, Sigma-Aldrich Co., USA). Three days after injection, blood samples were taken from the tail vein and glucose measurement by glucometer (Bionime model, Taiwan), the rats with fasting blood glucose levels of ≥ 300 mg/dl were acknowledged as diabetic. To confirm diabetes as chronic, the animals were housed for two weeks (4).

### Experimental design

The rats were randomly divided into six experimental groups, (*n* = 6) each consisting of six male rats including: 1 (Control), received a single dose of citrate buffer (0.5 ml) and two weeks later 0.5 ml of distilled water given daily; 2 (LC), a single dose of citrate buffer given and two wk later, received 100 mg/kg of LC (Sigma, C0283-5G, USA) (dissolved in distilled water) daily; 3 (Diabetic), two wk after STZ injection and confirmed diabetes, received a dose of distilled water daily; 4 (Diabetic +LC 50), two wk after confirmed diabetes, given 50 mg/kg of LC daily; 5 (Diabetic + LC 100), two wk after confirmed diabetes, received 100 mg/kg LC daily and 6 (Diabetic + LC 200 mg/kg), two wk after confirmed diabetes, given 200 mg/kg LC daily (11). All treatments were applied as Ip and continued for 48 days (according to the spermatogenesis period in rats). Blood glucose was measured in the first, middle, and last treatment period in the fasting rats. At the end of each experimental period, the rats were weighed under diethyl ether anesthesia, their left testis and epididymis were rapidly removed. The proper testis weight was measured (by R & D of 0.0001) and their relative body weight was estimated. The testis fixed in 10% formalin for histological study. The caudal part of epididymis was minced with scissors and put into small Petri dish containing 1 ml of Ham's F10 medium incubated at 37°C for 20 min in order to let spermatozoa swim out of the epididymal tubules in estimate spermatic parameters.

### Sperm analysis

Epididymal sperm analysis, including the motility, viability, count, and abnormal morphology of sperm were done as the previous studies indicated (11, 12).

### Sperm nuclear maturity

At the stage of spermiogenesis in the chromatin quality core, protamine is placed instead of histone. This replacement is highly important in sperm density and stability. The histone protein has a large number of lysine amino acids reacting with acidic colors such as aniline blue and turns blue. Therefore, in staining aniline blue, the immature sperms turn dark blue because of the presence of histones, while the healthy sperms are less affected by staining and are seen as pale. Briefly, the prepared spermatozoa were spread onto glass slides and allowed to dry at laboratory temperature. The smears were fixated in 70% alcohol for 10 min and finally were stained by aniline blue. At least 100 sperm cells per slide were evaluated using a light microscope at ×1000 magnification and the percentage of mature sperm in each animal was calculated (12).

### Sperm DNA integrity

Acridine Orange (AO) staining was performed to estimate the sperm DNA integrity denaturatin. Briefly, the sperm smears were dried at the air and fixed in a Carnoy's fixative (methanol/acetic acid, 3:1) at 4°C for 14 hr. The slides were stained for 8 min with freshly prepared AO (19% AO solution in citrate phosphate). Washed with distilled water and dried in air. The slides were examined on the same day using an immunofluorescence microscope (Zeiss Company, Germany) at ×1000 magnification. The green-headed sperms were marked to double-strand DNA integrity or healthy DNA integrity, and the spermatozoa with yellow or redhead were considered as single-stranded DNA integrity or denatured DNA integrity. On each slide at least 100 sperms were evaluated, the percentages of single-stranded DNA integrity and double-stranded sperm were evaluated and compared among the groups (13).

### Histopathological assay

The left testis was removed and fixed in alcoholic formalin 10% (Bouin fixative) for 24-48 hr, then processed into an automatic tissue processor system (SCILAB, England). Then tissue blocks were prepared on paraffin block-making Tissue Embedding Center (SCILAB, England). The 4-μm thick sections were obtained using a rotary microtome (Leica Model RM 2145, Germany). The prepared slides were stained using hematoxylin-eosin method for microscopic examination (11, 12).

### Morphometric study

Seminiferous tubules diameter and germinal epithelial height of the 50 round or nearly round cross-sections of seminiferous tubules using an ocular micrometer of light microscopy (Nikon, Japan) were randomly measured in each animal and their means were calculated. The germinal epithelial thickness was evaluated from the spermatogenic cells on the basement membrane through the sidelines cells of the tubules lumen (12).

### Spermatogenesis assessment

To evaluate spermatogenesis, at least 100 seminiferous tubules were examined in each animal and the quality of spermatogenesis in each tubule was scored according to the maturity of germ cells in the seminiferous tubules by Modified Johnsen's score system. Then the sum of all scores was divided by the total number of seminiferous tubular sections (12).

### Tunel assay

The percentage of apoptotic cells in testes was identified by Tunel assay, using an in situ detection kit (Roche Insitu Cell Death Detection Kit, Germany) according to the manufacturer's instructions (14).

### Ethics consideration

All animal experimentation protocols were carried out under the supervision of the Ethics Committee of Mazandaran University of Medical Sciences (Code no: IR.MAZUMS.Rec.96.2460).

### Statistical analysis

Data were analyzed using Graph Pad Prism 6.07 software. The results were expressed as Mean ± SD. Inter-group comparisons were performed with one-way analysis of variance (ANOVA) and Tukey's post hoc test; p < 0.05 was considered statistically significant.

## 3. Results

### Effects of LC on body weight, blood glucose, and testis weight

As shown in Table I, diabetes significantly decreased the body weight, but more significantly in the LC groups (p < 0.001). In the diabetic groups, the final glucose level was significantly higher than of the primary blood glucose, and LC treatment significantly improved blood glucose level (p < 0.01). In the diabetic group, the relative testis weight (gonadosomatic index) significantly decreased compared to the control group (p < 0.001), while the diabetic groups receiving LC revealed a significant increase in a dose-dependent manner (p < 0.001).

**Table 1:** Effect of L-carnitine (LC) on body and testis weights, a serum glucose level of adult male Wistar rats in control and treated groups


	**Control**	**L-carnitine**	**Diabetic**	**Diabetic + LC 50**	**Diabetic + LC 100**	**Diabetic + LC 200**
First body weight (g)	235.0 ± 11.83	241.7 ± 10.8	244.7 ± 6.05	242.2 ± 9.9	242.0 ± 12.85	245.8 ± 13.2
Last body weight (g)	271.8 ± 10.7${}^{\text{a}***}$	227.5 ± 10.37	214.7 ± 11.35${}^{\text{a}**}$	210.8 ± 12.81${}^{\text{a}***}$	196.7 ± 12.52${}^{\text{a}***}$	184.2 ± 14.63${}^{a***}$
First Glucose (mg/dl)	116.8 ± 9.4	119.2 ± 6.6	395.8 ± 55.3	398.3 ± 50.5	392.5 ± 82.0	396.7 ± 44.6
Mid-range Glucose (mg/dl)	118.0 ± 5.7	119.0 ± 10.6	475.0 ± 45.1	397.5 ± 49.9	366.7 ± 78.9	361.7 ± 41.6
Last Glucose (mg/dl)	118.5 ± 6.6	117.5 ± 10.8	530.0 ± 40.9${}^{\text{b}***}$	393.3 ± 50.5	348.3 ± 64.0	318.3 ± 26.5${}^{\text{b}**}$
Absolute testis weight (g)	1.42 ± 0.15	1.45 ± 0.14	1.17 ± 0.03${}^{\text{c}**}$	1.27 ± 0.09	1.33 ± 0.12	1.50 ± 0.10${}^{\text{d}***}$
Relative testis weight (per BW, %)	182.9 ± 12.26	186.2 ± 13.58	127.0 ± 12.21${}^{\text{e}***}$	135.1 ± 13.62	155.7 ± 16.68${}^{\text{f}*}$	162.3 ± 18.14${}^{f**}$
Note: The data are expressed as mean ± SD. Statistical significance is represented as follows: (*p < 0.05, **p < 0.01, ***p < 0.001)						
${}^{\text{a}}$: compared Last body weight with First body weight; ${}^{\text{b}}$: compared Last serum Glucose level with First Glucose level; ${}^{\text{c}}$: compared testis weight of rat in diabetic group with control group; ${}^{\text{d}}$: compared testis weight of rat in different groups with diabetic group; ${}^{\text{e}}$: compared Relative testis weight of rat in diabetic group with control group; ${}^{\text{f}}$: compared Relative testis weight of rat in different groups with diabetic group. LC: L-carnitine						

### Sperm examination findings

**Figure 1:**
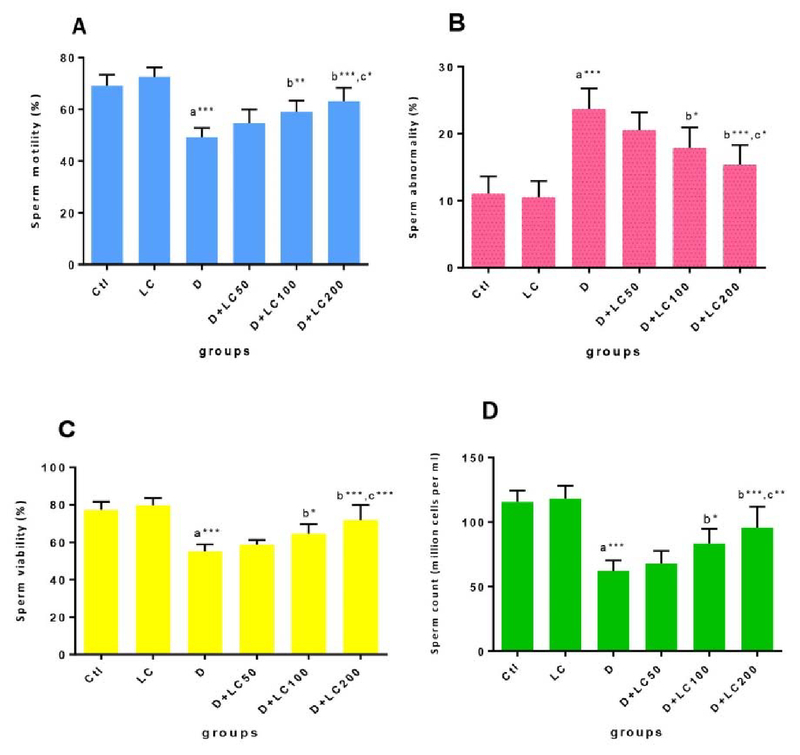
Effect of L-carnitine (LC) on sperm parameters in control and treated groups. The data are expressed as mean ± SD. Statistical significance is represented as follows: (*p < 0.05, **p < 0.01, ***p < 0.001). ${}^{\text{a}}$: compared with the control group; ${}^{\text{b}}$: compared with the diabetic group; ${}^{\text{c}}$: compared doses of LC 200, 100 with LC50. Ctl: Control group; LC: L-carnitine group; D: Diabetic group.

The sperm parameters' results in Figure 1 indicate that diabetes significantly reduced the sperm count, motility, viability, and increased the number of abnormal sperm (p < 0.001). While LC reduced diabetes-induced damage (p < 0.001) significantly and dose-dependent increased the count, motility, viability but reduced the abnormality of sperm. The analysis of sperm maturation and sperm DNA integrity are presented in Table II. Diabetes reduces the number of mature sperms and increases the percentage of single-stranded DNA integrity sperms (p < 0.001), while LC improved significantly (p < 0.001) the sperm maturation and the percentage of the double-stranded DNA integrity and healthy sperms. Comparing the sperm parameters didn't reveal any significant difference in groups 1 and 2.

### Histopathological findings 

**Figure 2:**
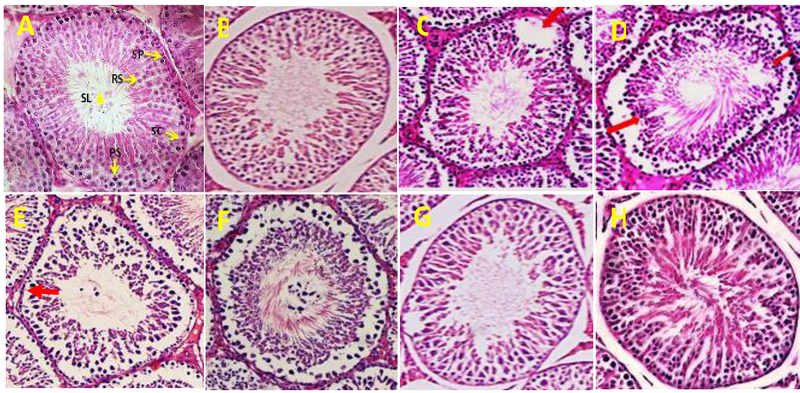
(A) Normal testis of the control group, (notice the normal and mature spermatozoa in seminiferous tubule lumen (SL), spermatogonia cell (SP), primary spermatocyte (PS), round spermatid (RS), Sertoli cell (SC)). (B) Testis in L-carnitine (LC) group. (C)-(E) Testis in the diabetic group showing degeneration, germinal epithelium height reduction and incomplete spermatogenic series (Up-down arrow), disruption of the sperm cells' first layer of germinal epithelium (red arrows), Vacuolation (thick arrow). (F), (G), and (H): Improvement of spermatogenesis of diabetic rats treated with LC. (H&E, ×400).

In the histopathologic study of the seminiferous tubules cross-sections structure in groups 1 and 2, the seminiferous tubules were slightly spaced apart and the space between them was filled by a small volume of interstitial tissue and Lydic cells. The germinal epithelial cells of these tubules were more numerous and more diverse, the Sertoli cells and all spermatogenesis cell lines, such as spermatogonia, primary spermatocytes, and spermatid, were visible, and a large number of spermatozoids exist in these tubules' lumen. However, compared with the control group in the diabetic group's seminiferous tubules, the disruption of the sperm cells' first layer (detachment of spermatogenic cells), deformation and seminiferous tubules' decreased congestion and the seminiferous tubules' structure's disorganization were observed. Also, the degenerated germinal epithelium cells and reduced number of spermatozoids in tubule lumen and vacuolation (the cavities formed in the germinal epithelium), as well as desquamation and exfoliation (the loss of part of the epithelium in the tube's lumen) were clearly observed. Using LC significantly decreased the diabetic-induced histopathologic and spermatogenesis complications (Figure 2).

### Effect of LC on seminiferous tubules diameter, germinal epi-thelium's height, and Johnsen's scores

Seminiferous tubules' diameter, germinal epithelium's height, and Johnsen's scores in the cross-sections of seminiferous tubules for all the groups are shown in Table III. In comparison with group 1, diabetes reduced these parameters significantly (p < 0.001), while in the animals treated with LC, these parameters mean was significantly higher than that of the group 3 (p < 0.001).

### Effect of LC on spermatogenic cells' apoptosis

**Figure 3:**
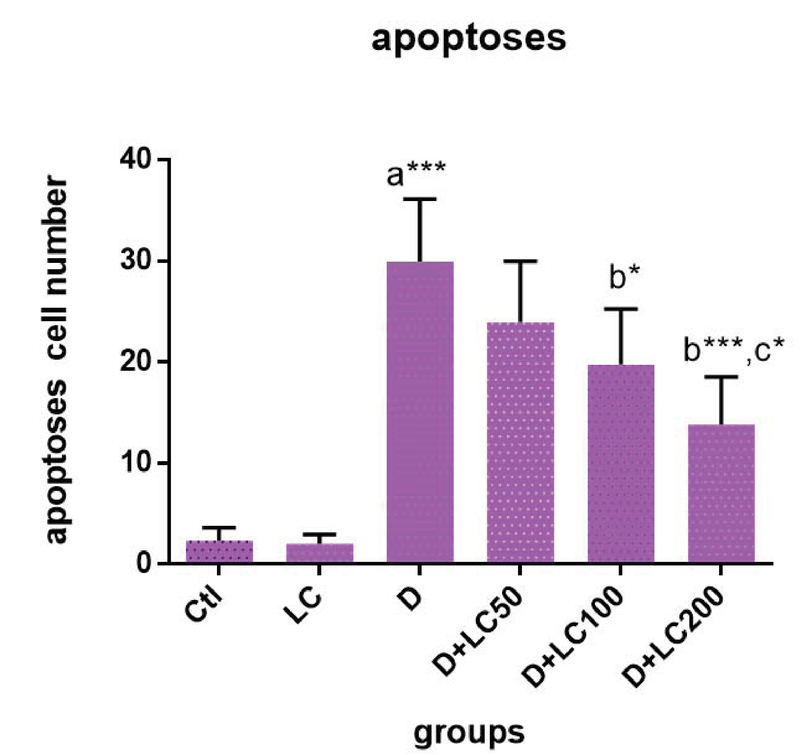
Effect of L-carnitine (LC) on spermatogenic cells' apoptosis in control and treated groups. The data are expressed as mean ± SD. Statistical significance is represented as follows:(*p < 0.05, ***p < 0.001). ${}^{\text{a}}$: compared with control group, ${}^{\text{b}}$: compared with diabetic group, ${}^{\text{c}}$: compared doses of LC 200, 100 with LC50. Ctl: Control group; LC: L-carnitine group; D: Diabetic group.

**Figure 4:**
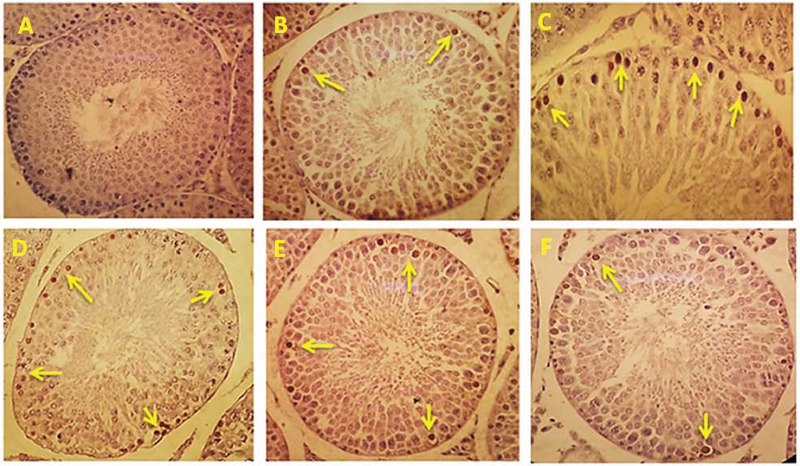
Effect of L-carnitine (LC) on spermatogenic cells' apoptosis in control and treated groups. Apoptotic cells turning dark brown (yellow arrow). (A) Control group, (B) LC group, (C) Diabetic group, (D) Diabetic+LC 50, (E) Diabetic + LC 100, (F) Diabetic + LC 200 (Tunnel staining, ×400).

The evaluation of rat testis apoptotic index is given in Figures 3 and 4. The apoptotic cells affected by the Tunnel color and turning dark brown are clearly visible in the early stages of spermatogenesis. In comparison with group 1, diabetes significantly increased the apoptotic cells number, while in the groups 4, 5, and 6, the apoptotic cells' number was significantly (p < 0.001) less than that of group 3.

**Table 2:** Effect of L-carnitine (LC) on sperm maturity and sperm DNA quality of adult male Wistar rats in control and treated groups


**Group**	**Control**	**L-carnitine**	**Diabetic**	**Diabetic + LC50**	**Diabetic + LC100**	**Diabetic + LC200**
Sperm maturity (%)	85.58 ± 4.42	86.35 ± 5.45	64.43 ± 4.46${}^{\text{a}}$***	66.28 ± 3.22	71.48 ± 3.37	79.20 ± 8.72${}^{\text{b}}$***,${}^{\text{c}}$**
DNA integrity (%)	6.0 ± 1.78	5.66 ± 1.36	15.33 ± 1.86${}^{\text{a}}$***	14.50 ± 1.51	12.83 ± 1.47	10.67 ± 1.36${}^{\text{b}}$***,${}^{\text{c}}$**
The data are expressed as mean ± SD. Statistical significance is represented as follows: (**p < 0.01, ***p < 0.001) LC: L-carnitine ${}^{\text{a}}$: compared with control group; ${}^{\text{b}}$: compared with diabetic group; ${}^{\text{c}}$: compared doses of LC 200, 100 with LC50						

**Table 3:** Johnsen's score, seminiferous tubule diameter and germinal epithelium height in different groups


**Group**	**Control**	**L-carnitine**	**Diabetic**	**Diabetic + LC50**	**Diabetic + LC100**	**Diabetic + LC200**
STD (µm)	285.7 ± 15.81	287.5 ± 15.43	249.9 ± 10.86${}^{\text{a}}$***	251.5 ± 13.06	256.1 ± 12.34	10.94 ± 261.5${}^{\text{b}}$***,${}^{\text{c}}$**
EGH (µm)	92.10 ± 10.21	94.72 ± 10.10	54.10 ± 11.98${}^{\text{a}}$***	56.80 ± 11.37	59.80 ± 11.34	64.00 ± 14.29${}^{\text{b}}$***,${}^{\text{c}}$*
Johnson’s scores	9.19 ± 0.39	9.22 ± 0.53	6.06 ± 0.75${}^{\text{a}}$***	6.21 ± 0.70	7.39 ± 1.05${}^{\text{b}}$*	8.08 ± 0.83${}^{\text{b}}$***,${}^{\text{c}}$**
Note: The data are expressed as mean ± SD. Statistical significance is represented as follows: (*p < 0.05, **p < 0.01, ***p < 0.001) ${}^{\text{a}}$: compared with control group; ${}^{\text{b}}$: compared with diabetic group; ${}^{\text{c}}$: compared doses of LC 200, 100 with LC50. STD: seminiferous tubule diameter; GEH: Germinal epithelium height; LC: L-carnitine						

## 4. Discussion

The purpose of the present study was to investigate the effects of LC on the reproductive parameters of STZ-induced diabetic adult male Wistar rats. Our finding has shown that STZ has caused diabetes, increased blood glucose, and decreased body and testis weight. The obtained data are consistent with the other studies (15, 16). We noticed that the use of different concentrations of LC reduced body weight and decreased blood glucose level. It agrees with the other findings (17, 18). Salmanoglu and co-worker investigating the protective effect of LC on gonadotocix testis suggested that LC consumption reduced body weight and decreased blood glucose levels (19). L-carnitine facilitates beta-oxidation of high-chain fatty acids that are effective in weight loss through reducing fat tissue. Also, LC by increasing the release of glucose into the cell and activating some enzymes of the glycolysis pathway plays an important role in reducing blood glucose levels (17).

We found that diabetes results in the spermatogenic cells' first layer disruption, reduced density, and increased irregularity in the seminiferous tubules' structure, reduced seminiferous tubule diameter and germinal epithelium height and degenerated spermatogenesis cell lines, vacuolization, and exfoliation; even spermatozoids number reduces in the seminiferous tubules lumen. In group 6, the administration of LC 200 mg/kg significantly increased the seminiferous tubules' density and diameter, germinal epithelium height (p < 0.001), and improved the spermatogenesis cells' first layer's disruption, vacuolization, and exfoliation. Sazgara and co-worker revealed that diabetes-induced disruption of the spermatogenesis cells' first layer deformed the seminiferous tubules and reduced germinal epithelium thickness; treatment with LC improved the seminiferous tubules' diameter and germinal epithelium's thickness, compared to the other groups (20). Concerning LC-induced effect on the seminiferous tubule's diameter, it could be said that LC's protective and anti-oxidant role against free radicals reduces the oxidative stress, accelerating spermatogenesis cells' differentiation and increasing sperm release from the seminiferous tubule luminal surface (20).

We also found that diabetes reduced the count, motility, and viability of sperm and increased the number of abnormal and immature sperms, which agrees with the findings of other studies (21, 22). In the present study, in groups 5 and 6 in a dose-dependent manner, the count, motility, and viability of the sperm increased significantly, and the abnormality and immaturity of sperm decreased. These results correspond to the findings of other relevant studies (21, 23). Possible mechanisms for the effect of LC on spermatogenesis can be justified in several ways: 1) the mitochondrial inner membrane is impermeable to long-chain fatty acids, and fatty acids to activate, must be coupled with acetyl coenzyme A prior to passing through the mitochondrial membrane. In turn, acetyl coenzyme A molecules require LC as a cofactor. Then LC by facilitating lipid metabolism supplies the energy required for sperm motility (24); 2) since epididymis is the site of maturation store of sperm, the place for sperm to get motility, and the highest LC level is in the epididymitis. Therefore, in many individuals with reduced count and motility of sperm due to idiopathic reasons, the administration of LC plays a decisive role in improving sperm parameters and has a positive effect on the epididymis environment, which affects the quality of sperm, resulting in increasing sperm count (25); 3) it is probable the LC by its antioxidant properties protects the sperm membrane against free radicals and oxidative stress phenomenon. This property may be due to LC's potential to absorb free iron ions, to inhibit superoxide ions production, and detoxification of hydrogen peroxide species. Studies have exhibited that increased free radicals and ROS accumulation in sperms exert adverse effects on the activity and fertility of sperm. Free radicals produced in diabetes can damage testis tissue and reduce sperm count. Therefore, it could be concluded that LC by reducing ROS, free radicals scavenger, and raising the antioxidant system prevents oxidative stress severity caused by diabetes and exerts beneficial effects on the motility and viability of sperm, prevents tissue changes in the testis and, through preserving the normal structure of the testis, improves the spermatogenesis process (26).

Another remarkable result of the present study is that diabetes significantly increases apoptotic cells' number in rat testis (p< 0.001) and using LC 100 mg/kg and 200 mg/kg as dose-dependent significantly reduced the risk of diabetic apoptosis in the rat testis. Several studies are consistent with our obtained data (27, 28). In diabetic patients, the apoptosis of spermatogenesis relies on several factors: 1) reducing the levels of essential hormones for spermatogenesis such as FSH, LH, and testosterone (29); 2) reducing the level of antioxidants such as glutathione (GSH) in the mitochondria of the sperm, which in turn increases the production of free oxygen radicals and the formation of membrane canals in mitochondria. The decrease in the antioxidants such as, GSH in the sperm mitochondria, in turn, increases the productions oxygen-free radicals and formation of membrane canals in mitochondria (30); 3) increases free radicals production because mammalian sperm cells due to their large lipids contain high levels of unsaturated fatty acids, are a good site to produce free radicals due to lipid peroxidation (31); 4) testicular cells have a high metabolism due to sequential divisions, and results in more free radicals production; 5) due to increased blood glucose in diabetic patients, hemoglobin glycosylated (HbA1c) blood levels go up. The final products of this glycosylation are ROS, which can induce cells apoptosis (32). Therefore, increasing free radicals production and the reducing antioxidant defense system cause these radicals' accumulation in the cell, which ultimately affects the cell activity, leading to membrane channels' formation in mitochondria and activation of caspases 9 and 3. These factors ultimately exacerbate apoptosis in the testis (33); (6) a wide range of free toxic compounds increase mitochondrial membrane's permeability, one of which is the presence of free fatty acids surrounding mitochondria. Data indicate that the free-chain fatty acids accumulation around mitochondria may lead to mitochondrial membrane permeability. Due to the mitochondrial membrane depolarization and membrane canals formation, the mitochondrial membrane permeability changes. This releases cytochrome C from mitochondria and forms apoptosomal complexes, and ultimately, activates the apoptotic mechanism in cells by activating caspase cascades (34). L-carnitine likely affects cells apoptosis in several ways: Firstly, LC effectively inhibits the mitochondrial membrane depolarization and permeability, resulting in apoptosis by transferring the accumulated long-chain fatty acid surrounding mitochondria. According to this hypothesis, the mitochondrial membrane permeability and cell apoptosis are performed by a balance between lipid and LC in and around the mitochondria membrane (28). Secondly, LC can inhibit the activity of caspases and prevent apoptosis. Thirdly, LC, as a potent non-enzymatic antioxidant, can reduce apoptosis via increasing antioxidant defense and reducing the production of free radicals (35).

## Conclusion

Our findings indicate that LC consumption in diabetic rats significantly improves the relative testis weight, blood glucose levels, and significantly increases the count, motility, and viability and reduces the abnormality of sperm, which is dose-dependent. In addition, LC consumption significantly increases the sperm maturity and percentage of sperms with double-stranded and healthy DNA integrity which ultimately improves the sperms parameters and significantly reduces the complications of DM on testicular tissue, seminiferous tubules, and apoptosis in spermatogenic cells. However, further studies are recommended to achieve the most effective dose of LC in DM..

## Conflict of Interest

The authors declare no conflict of interests in the present study.
